# Project-Based Learning in Medical Physiology: Effects on Knowledge Gain and Student Perceptions

**DOI:** 10.7759/cureus.117712

**Published:** 2026-10-09

**Authors:** Aparna Menon, K. Venkatesh, Kanthasamy Kiran, R. Premaraja, Boddepalli Abhiram, Monica P. V., R. Divya

**Affiliations:** 1 Physiology, Dhanalakshmi Srinivasan Medical College and Hospital, Perambalur, IND; 2 Physiology, Swamy Vivekanadha Medical College Hospital and Research Institute, Namakkal, IND; 3 Physiology, Dhanalakshmi Srinivasan Group of Institutions, Perambalur, IND; 4 Physiology, Melmaruvathur Adhiparasakthi Institute of Medical Sciences and Research, Melmaruvathur, IND; 5 Physiology, Trichy SRM Medical College Hospital and Research Centre, Trichy, IND

**Keywords:** competency-based medical education (cbme), innovative teaching strategies, pedagogical innovation, physiology education, practical learning, project-based learning, research interest, student engagement, teaching-learning tool, undergraduate medical education

## Abstract

Background: Although project-based learning (PBL) is well-established in advanced medical curricula, evidence supporting its implementation in first-year undergraduate programs remains limited. Early research exposure may enhance student engagement.

Objective: This study evaluated whether structured PBL in muscle physiology was associated with improvements in subject knowledge acquisition among first-year MBBS students.

Methods: An observational study was conducted at a single medical institution in Tamil Nadu, India, from July to August 2023. Formal institutional ethics approval was obtained prior to participant recruitment. Of 250 eligible first-year MBBS students, 40 consented to participate; five subsequently withdrew citing curriculum workload concerns, yielding 35 participants (30 females, five males; mean age 18.35 years). Students underwent pre-assessment comprising 10 short-answer questions on muscle physiology (maximum score 20) and were randomized to 10 project groups via lottery. Students conducted investigations on exercise physiology over three to four hours weekly for one month. Post-assessment utilized identical questions. Thirty-two students completed a structured feedback questionnaire (six Likert-scale items, 1-5 scale) and provided open-ended commentary. Assessment scores were compared via paired t-testing and Shapiro-Wilk testing. Pearson correlation assessed satisfaction-knowledge associations. Qualitative analysis employed thematic coding by two independent raters using predefined and emergent themes with inter-rater agreement assessment.

Results: Post-assessment scores (12.588 ± 2.943) significantly exceeded pre-assessment scores (5.265 ± 2.89), yielding a mean improvement of 7.324 ± 2.446 points (t(34) = -17.952, p < 0.001, Cohen's d = -3.034; 95% CI (-3.821, -2.239)). This large effect size must be interpreted cautiously given the observational design, self-selected sample, identical assessment questions, and potential testing-practice effects. Post-hoc power analysis confirmed an achieved power of 0.99. The mean satisfaction score was 22.563 ± 3.519 out of 30. No statistically significant association existed between satisfaction and knowledge improvement (r = 0.082, p = 0.657). Qualitative analysis demonstrated predominantly favorable responses, with 26 of 35 students (74.3%) expressing interest in future research participation.

Conclusion: Structured PBL was associated with substantial knowledge improvement and positive student perception among participating students. However, the single-group observational design, self-selected sample, and identical assessments preclude causal inference regarding PBL's effectiveness. Larger comparative randomized trials are necessary to establish whether PBL produces educational benefits relative to standard instruction and whether effects persist over time.

## Introduction

Contemporary medical education emphasizes the development of not only comprehensive subject knowledge but also higher-order cognitive competencies, critical appraisal skills, and lifelong learning capacity [1]. Achieving these multifaceted educational objectives necessitates pedagogical innovation extending beyond traditional didactic lectures [2]. Research engagement has become increasingly recognized as an important component of contemporary medical education. Structured introduction of research projects offers an accessible pathway to research methodology while fostering intellectual development and cultivating scientific inquiry [3]. Such early exposure to research processes may enhance student engagement and deepen subject comprehension.

Notwithstanding the recognized importance of research participation, first-year medical students frequently perceive research as an undertaking beyond their current capability or of limited relevance to their immediate studies [4]. This perception represents a significant obstacle to early research engagement. Strategic introduction of circumscribed, structured research projects tailored to first-year students - distinct from the more extensive investigations characterizing later curricula - may effectively address this barrier while fostering research interest and demonstrating the accessibility of research methodology [5]. Such initiatives expose students to multidimensional aspects of scientific inquiry, encompassing research literacy, problem-based reasoning, and autonomous learning.

Contemporary evidence from Indian medical education indicates that integrating research into undergraduate medical education is aligned with competency-based medical education (CBME) frameworks and National Medical Commission (NMC) competencies [6]. The NMC actively encourages all cohorts, including first-year students, to cultivate research interests across disciplinary areas and to develop research competencies as part of their medical training. Accumulating evidence indicates that early research involvement augments scientific identity, deepens disciplinary comprehension, enhances intrinsic motivation, and fosters professional empathy [7,8].

However, substantial barriers to initiating research participation persist among first-year students, principally limited understanding of research scope and its connection to clinical practice [9,10]. Passive provision of opportunities frequently proves insufficient to stimulate meaningful engagement among uncertain early-career students. Consequently, structured group research projects integrated within first-year physiology, anatomy, or biochemistry curricula may serve as an accessible portal to research methodology while simultaneously strengthening subject mastery [11].

Beyond its research-exposure benefits, project-based learning (PBL) has demonstrated considerable efficacy in enhancing subject comprehension and student engagement in medical education [12]. This pedagogical approach cultivates sophisticated learning processes encompassing self-directed inquiry, collaborative problem-solving, and peer-mediated learning [13]. By establishing meaningful connections between abstract theoretical concepts and practical applications, learners develop substantive conceptual frameworks [14,15]. Notwithstanding substantial international literature evaluating PBL within later medical curricula and allied health professions, evidence from Indian first-year undergraduate medical programs remains scarce.

The present investigation evaluated whether structured PBL in muscle physiology could enhance knowledge acquisition and cultivate research interest among first-year MBBS students with limited prior exposure to such pedagogical approaches. We hypothesized that early implementation of research-based learning would demonstrate associations with improved knowledge outcomes and foster a perception of research as integral, accessible, and worthwhile within medical training.

## Materials and methods

Study setting, design, and participants

This observational, descriptive study was conducted within the Department of Physiology at Dhanalakshmi Srinivasan Medical College and Hospital, Siruvacharur, Perambalur district, Tamil Nadu, India, from July to August 2023 (a one-month period). Formal institutional ethics approval was obtained on 01 July 2023 prior to participant recruitment and data collection. Student recruitment commenced in early July 2023. The PBL intervention and investigations were conducted from mid-July through August 2023, culminating in student presentations on 6 August 2023 and immediate post-assessment and feedback collection during the final week of August 2023. All 250 first-year MBBS students from a single cohort (all aged ≥18 years) were invited to participate in structured supplementary PBL on muscle physiology, conducted independently of the regular curriculum. Recruitment was exclusively merit-based, informed by genuine student interest. All participating students received comprehensive information regarding the anticipated benefits of research participation. Of those expressing initial interest (n = 40), and following provision of written informed consent, five subsequently withdrew prior to project commencement, attributing their decision to anticipated workload conflicts with existing curricular obligations. The final study cohort comprised 35 participants (86% female, 14% male; mean age 18.35 ± 0.51 years).

Ethical approval and informed consent

Institutional ethics approval was granted by the Institutional Ethics Committee for Human Studies (IECHS) and Institutional Research Committee (IRC) of Dhanalakshmi Srinivasan Medical College (approval number IECHS/IRCHS/No.314; approval date 01 July 2023). Formal institutional ethics approval was obtained prospectively prior to participant recruitment and data collection. All 40 initially consenting students provided written informed consent prior to study participation. Participation was entirely voluntary, with no academic incentives (supplementary marks, extra credit, or grade modification) offered. No adverse consequences ensued from non-participation or study withdrawal.

Inclusion and exclusion criteria

Inclusion criteria comprised: (1) current enrollment as a first-year MBBS student; (2) age ≥18 years; (3) willingness to provide informed written consent. All 250 first-year students (n = 250) met these criteria. Formal exclusion criteria were not pre-specified. Operationally, students unwilling to provide consent were not enrolled. Subsequent to written consent, five students self-selected withdrawal upon recognizing anticipated curriculum integration challenges.

PBL intervention

The intervention was scheduled following completion of the muscle physiology unit within the institutional CBME framework. Students were allocated three to four hours weekly for project-related activities conducted outside standard curriculum hours. Ten researcher-designed investigations on muscle physiology were developed, considering students' pre-existing knowledge and available institutional equipment. Students were organized into 10 groups (three to five members each); group-project assignments were determined via lottery randomization to ensure unbiased allocation.

Investigations addressed diverse exercise physiology phenomena: (1) fatigue during isotonic versus isometric exercise ± venous occlusion; (2) post-exercise recovery kinetics; (3) cardiovascular and respiratory adaptation to exercise; (4) neurocognitive effects of physical activity; (5) calculation of maximal oxygen consumption and metabolic equivalents; (6) skeletal muscle and exercise types; (7) muscle strength-endurance relationships; (8) metabolic effects of different exercise intensities; (9) physiological responses to submaximal exercise; and (10) exercise capacity assessment in health and disease. Each group received structured training encompassing equipment operation, standardized data collection protocols, and computational procedures relevant to their assigned investigation. Faculty supervision was provided through weekly contact hours, utilizing both directive (guidance on protocol adherence) and facilitative (encouraging student autonomy in problem-solving) approaches.

Following data acquisition, students underwent structured instruction in fundamental statistical analysis, encompassing descriptive statistics, variability measures, and data visualization (tabular, bar diagram, and pie chart representations). Each group prepared a formal written report (approximately five to seven pages) adhering to scientific manuscript conventions and delivered oral findings to an audience comprising departmental faculty and peer groups (approximately 15 minutes presentation per group).

Assessment and measurement instruments

Knowledge Assessment

Pre-intervention assessment comprised 10 open-ended short-answer questions addressing muscle physiology concepts (Appendix A). Each item carried two marks, yielding a maximum possible score of 20. The assessment was administered prior to project commencement. An equivalent post-intervention assessment utilized identical items. Following post-assessment completion, students performed self-evaluation utilizing provided answer keys. Final scores underwent investigator verification to minimize assessment bias. Scoring was performed independently by two raters (lead and corresponding authors) using a predetermined rubric with explicit criteria: 0 marks = no response or entirely incorrect; 1 mark = partially correct or incomplete answer; 2 marks = correct and complete answer. Discrepancies between raters were resolved through discussion; final scores underwent verification by a third faculty member.

Satisfaction and Feedback Measurement

Student satisfaction was quantified using a researcher-developed six-item questionnaire employing a five-point Likert scale (1 = minimal, 5 = maximal) (Appendix B). Items assessed: overall project experience; subject knowledge acquisition; practical competency development; subject interest cultivation; research methodology interest; and interest in future research projects. This instrument underwent systematic development and peer-review validation by a panel of four faculty members (two physiologists, two medical educators) prior to implementation, who assessed clarity, relevance, and comprehensiveness of items. Revisions based on reviewer feedback were incorporated prior to pilot administration. Additionally, students provided open-ended commentary regarding their project experience.

Qualitative Analysis of Open-Ended Feedback

Open-ended responses were coded inductively using thematic analysis by two independent coders (lead author and one research team member). A hybrid coding approach was employed: predefined themes were derived from the six questionnaire domains (knowledge acquisition, practical competency, subject interest, research interest, curriculum integration, overall experience) and combined with emergent themes identified from the responses themselves. Both coders independently reviewed all open-ended responses and categorized them thematically; inter-rater agreement was assessed using Cohen's kappa (κ = 0.338), indicating fair agreement [16]. This fair-level agreement reflects the exploratory nature of the qualitative analysis and the inherent subjectivity in categorizing open-ended student feedback. Coding disagreements were resolved through consensus discussion between the two coders to establish final thematic categorization, a standard approach in exploratory qualitative research. The qualitative findings should be interpreted within the context of this fair inter-rater reliability. Final themes and representative quotations were verified against original responses to ensure accuracy and fidelity.

Research Interest Assessment

Qualitative analysis of open-ended verbal feedback from all 35 participants identified patterns of research interest. Of the 35 students providing verbal feedback, 26 (74.3%) expressed unconditional interest in future research participation, while nine (25.7%) indicated conditional interest contingent upon project topic selection. Analysis of open-ended comments revealed that students emphasizing conditional interest frequently stated that their willingness to pursue research depended on project topic selection. Among the 32 students completing the structured feedback questionnaire, Item 6 (research interest on a five-point Likert scale) yielded a mean response of 3.78 ± 1.23, complementing the qualitative findings.

Statistical analysis

Descriptive statistics summarized participant characteristics and assessment outcomes as mean ± standard deviation (SD). Distributional normality of assessment and satisfaction scores was evaluated via Shapiro-Wilk testing. Within-subject knowledge change was assessed using paired-samples t-testing (n = 35). Post-hoc power analysis was conducted using G*Power (Ver. 3.1 Heinrich-Heine-Universität Düsseldorf, Düsseldorf, Germany) [17] to determine achieved statistical power for the observed effect size (Cohen's d = -3.034). A priori power assumption was a medium effect size (f = 0.25, α = 0.05, two-tailed) as appropriate for exploratory study design. The association between satisfaction ratings and knowledge improvement was quantified through Pearson product-moment correlation analysis (n = 32; feedback completion rate 91.4%). All statistical procedures were implemented using Microsoft Excel (Microsoft Corp., Redmond, WA, USA) and JASP (version 0.16.4.0; JASP Team, University of Amsterdam, Amsterdam, Netherlands). Bilateral p-values < 0.05 were considered statistically significant.

## Results

Participant characteristics and study flow

Of 250 eligible first-year MBBS students, 40 (16%) expressed interest and provided informed consent. Five students (12.5% of the registered cohort) subsequently withdrew prior to commencement, citing anticipated workload pressures. The analyzable cohort comprised 35 participants (30 females (85.7%), five males (14.3%)). The mean participant age was 18.35 ± 0.51 years. All participants completed both pre- and post-assessment procedures. Thirty-two participants (91.4%) returned completed feedback questionnaires. Demographic characteristics are presented in Table 1.

**Table 1:** Demographic and Recruitment Characteristics of Study Participants Percentages for initially registered, withdrew before the project, and final analyzable cohort are calculated with reference to the total eligible student population (n = 250). Percentages for sex and feedback questionnaire completion are calculated with reference to the final analyzable cohort (n = 35). SD: standard deviation

Characteristic	Value
Total eligible students	250
Initially registered, n (%)	40 (16.0)
Withdrew before project, n (%)	5 (12.5)
Final analysable cohort, n (%)	35 (14.0)
Female, n (%)	30 (85.7)
Male, n (%)	5 (14.3)
Mean age ± SD, years	18.35 ± 0.51
Feedback questionnaire completion, n (%)	32 (91.4)

Knowledge acquisition outcomes

Pre-intervention assessment demonstrated limited baseline knowledge, with a mean score of 5.265 ± 2.89 (range 1-16, 26.3% of maximum). Post-intervention scores improved substantially to 12.588 ± 2.943 (range 8-19, 62.9% of maximum). The mean absolute improvement was 7.324 ± 2.446 points (36.6% knowledge gain), indicating substantial knowledge consolidation. Paired-samples t-testing revealed highly significant within-subject knowledge improvement following PBL (t(34) = -17.952, p < 0.001). The effect magnitude was exceptionally large by statistical convention (Cohen's d = -3.034; 95% CI (-3.821, -2.239)).

However, this large effect size must be interpreted with considerable caution. The single-group pre-post observational design without a concurrent control group, use of identical assessment questions (introducing potential testing-practice effects and familiarity with question format), self-selected sample composition, and small sample size all suggest that the observed effect size may substantially overestimate the true intervention impact. The large effect likely reflects a combination of genuine knowledge consolidation from the project work, assessment practice effects, positive selection bias, and potentially other confounding factors rather than PBL intervention effects alone.

Post-hoc power analysis confirmed that the achieved statistical power for the observed effect size was 0.99 (99%), substantially exceeding the conventional adequacy threshold of 0.80 (80%). Assessment outcomes are detailed in Table 2.

**Table 2:** Knowledge Assessment Scores Before and After the Project-Based Learning Intervention The knowledge assessment comprised 10 short-answer questions, each carrying two marks, with a maximum possible score of 20. Knowledge improvement represents the post-assessment score minus the pre-assessment score. A paired-samples t-test demonstrated a statistically significant improvement in knowledge scores: t(34) = -17.952, p < 0.001; Cohen's d = -3.034 (95% CI, -3.821 to -2.239). Post-hoc power analysis confirmed achieved statistical power of 0.99 for the observed effect magnitude. SD: standard deviation; CI: confidence interval

Assessment Timepoint	Mean ± SD	Range (Min-Max)
Pre-assessment (n = 35)	5.265 ± 2.890	1-16
Post-assessment (n = 35)	12.588 ± 2.943	8-19
Knowledge improvement (n = 35)	7.324 ± 2.446	2-15
Pre-assessment (% of maximum score)	26.3	5-80
Post-assessment (% of maximum score)	62.9	40-95

Student satisfaction and feedback analysis

Among 32 students completing the satisfaction questionnaire, the mean satisfaction score was 22.563 ± 3.519 (range 14-29), representing 75.2% of the maximum possible score of 30. Pearson product-moment correlation analysis examined the relationship between satisfaction ratings and knowledge improvement magnitude, revealing no statistically significant association (r = 0.082, p = 0.657). This finding suggests that students' satisfaction with the project experience was independent of the magnitude of quantifiable knowledge gains. Qualitative analysis of open-ended feedback demonstrated predominantly positive commentary. Qualitative analysis of open-ended verbal feedback from all 35 participants identified patterns of research interest. Of the 35 students providing verbal feedback, 26 (74.3%) expressed unconditional interest in future research participation, while nine (25.7%) indicated conditional interest contingent upon project topic selection. Analysis of open-ended comments revealed that students emphasizing conditional interest frequently stated that their willingness to pursue research depended on project topic selection. Among the 32 students completing the structured feedback questionnaire, Item 6 assessed research interest on a five-point Likert scale, complementing the qualitative findings. Common evaluative descriptors included informative, intellectually stimulating, valuable, and knowledge-enhancing. Frequently cited suggestions for program enhancement included provision of additional specialized equipment, allocation of expanded time for statistical data analysis, facilitation of cross-group comparative reviews, and enhanced faculty mentorship. Student research interest categories are presented in Table 3.

**Table 3:** Research Interest Based on Qualitative Analysis of Open-Ended Feedback and Feedback Questionnaire Completion *Research interest categories were derived from qualitative analysis of open-ended verbal feedback collected from all 35 participants immediately following the intervention. Percentages are calculated based on the 35 participants providing verbal feedback (26 + 9 = 35). The feedback questionnaire (n = 32) was administered subsequently and included Item 6 assessing research interest on a five-point Likert scale as a complementary quantitative measure.

Research Interest Category	n	Percentage (%)
Unconditional interest in future research*	26	74.3
Conditional interest (topic-dependent)*	9	25.7
Total participants providing verbal feedback	35	100.0
Feedback questionnaire completion	32	91.4

Statistical test assumptions

Shapiro-Wilk testing confirmed that both pre-assessment and post-assessment scores approximated normal distributions (pre-assessment: W = 0.96, p = 0.22; post-assessment: W = 0.94, p = 0.09), supporting the appropriateness of paired-samples t-testing for primary analysis. The distribution of difference scores (improvement magnitude) similarly did not significantly deviate from normality (W = 0.95, p = 0.14).

## Discussion

Principal findings and interpretation

This observational investigation demonstrated that structured PBL was substantially associated with improved knowledge acquisition in muscle physiology among first-year medical undergraduates. The magnitude of improvement - quantified as a mean 7.3-point gain on a 20-point scale (36.6% increase), with a large effect size (Cohen's d = -3.034) by statistical convention - represents a notable change in short-term assessment performance. Notably, improvement occurred across the entire spectrum of baseline knowledge levels, suggesting that the project-based approach engaged students with heterogeneous baseline knowledge.

However, several factors prevent us from attributing this improvement specifically to the PBL intervention. Without a concurrent control group receiving standard instruction only, we cannot exclude alternative explanations, including knowledge consolidation through repeated assessment exposure, temporal maturation of students' understanding over the one-month period, exposure to the regular muscle physiology curriculum during this period, or testing-practice effects from identical pre- and post-assessment questions. The self-selected nature of participation (14% of eligible students) suggests that students who chose to engage in this activity may differ systematically in motivation, baseline knowledge, or learning strategies from their peers, differences that could themselves account for improved performance.

The marked dissociation between satisfaction ratings and knowledge improvement magnitude (r = 0.082, p = 0.657) warrants particular emphasis. This pattern suggests that students valued the project experience for reasons extending beyond immediate measurable learning outcomes. Potential explanations encompass exposure to authentic research processes, collaborative peer engagement, cultivation of practical competencies, and perceived career relevance.

Contextualization within existing literature

The present findings corroborate existing international literature demonstrating PBL's association with improved academic outcomes across diverse educational contexts. Investigations from Southeast Asian institutions have documented improvements in academic achievement, enhanced engagement, and strengthened interpersonal dynamics following project-based implementations [18-20]. European educational research similarly demonstrates associations between project-based approaches and enhanced performance across heterogeneous learner populations and learning styles [21].

Mechanisms underlying educational benefits

The mechanisms potentially underlying observed associations appear multifactorial and warrant investigation in controlled designs. Project-based pedagogy may enhance student agency, transitioning learners from passive knowledge recipients to active scientific investigators [21]. Small-group learning configurations may simultaneously augment interpersonal cohesion and individual accountability [22]. Authentic laboratory engagement may develop practical competencies while consolidating conceptual understanding [23]. Furthermore, project-based methodologies appear potentially effective in cultivating innovation, creativity, and sophisticated problem-solving capacities [24]. Faculty observations consistently document enhanced motivation and engagement in students undertaking project-based curricula [25]. However, without controlled comparison, we cannot determine whether these mechanisms operated in our setting or contributed to observed outcomes.

Implications for undergraduate medical education

These findings present several implications for contemporary undergraduate medical education, particularly within the Indian context, contingent on replication in larger controlled designs. First, the demonstration that first-year students can be engaged in meaningful disciplinary research suggests that conventional assumptions about temporal sequencing of medical curricula warrant reconsideration. This observation aligns with emerging pedagogical consensus suggesting that early research exposure may effectively address identified barriers to research participation in medical education.

Second, the substantial proportion of students expressing research interest following the intervention (74.3% unconditional, 25.7% conditional) suggests that strategically designed PBL may serve as a feasible mechanism for cultivating research interest from the curriculum's inception. However, expressed interest does not demonstrate research skill acquisition or predict actual research participation in subsequent years; longitudinal investigation is needed to examine whether such early engagement translates into sustained research involvement.

Third, the observed improvement in assessment scores alongside positive student feedback suggests that PBL was perceived favorably by participating students and was associated with demonstrated knowledge gains. Whether PBL constitutes an effective supplementary pedagogical modality requires evidence from larger controlled trials with diverse student populations and institutional contexts.

Study limitations

This investigation incorporates several significant methodological limitations warranting transparent acknowledgment. First, participants constituted a self-selected cohort representing only 14% of the eligible first-year population, introducing potential selection bias. Students expressing research interest or possessing particular academic inclinations, stronger baseline knowledge, or higher motivation may be substantially overrepresented relative to the broader student population. Any or all of these characteristics could independently or synergistically contribute to the observed knowledge improvement and favorable satisfaction ratings, making it impossible to isolate PBL's specific contribution. Consequently, findings cannot be generalized to the entire first-year cohort or to populations with different motivational or academic profiles.

Second, the investigation's observational design without concurrent control or comparator group precludes definitive causal attribution. Alternative explanations for observed knowledge improvements - including knowledge consolidation through repeated assessment exposure, temporal maturation, exposure to the regular muscle physiology curriculum during the study period, or concurrent independent study - cannot be definitively excluded. The magnitude of improvement (36.6% gain) renders testing-practice effects an implausible singular explanation; however, their contribution cannot be completely dismissed. The study employed identical assessment instruments for pre- and post-measurement, introducing this potential source of bias.

Third, the brief intervention duration (one month) and single-institution setting substantially constrain temporal and geographic generalizability. The 91.4% feedback questionnaire completion rate, while acceptable, suggests that three participants' experiences remain undocumented. Finally, the investigation incorporates no extended follow-up; persistence of knowledge gains or translation of expressed research interest into subsequent research participation remains unknown. Without longitudinal data, we cannot determine whether the effects observed immediately post-intervention persist over time or whether students' expressed interest translates into actual research engagement in subsequent years.

The qualitative analysis, while providing valuable insights into student feedback, demonstrated fair inter-rater agreement (κ = 0.338). While this level is technically acceptable in exploratory qualitative research, it reflects the inherent subjectivity in categorizing open-ended responses and suggests that future larger-scale studies would benefit from more structured qualitative methods, including predetermined coding frameworks, multiple independent coders, and formal qualitative analysis software (e.g., NVivo or ATLAS.ti).

## Conclusions

This observational study demonstrates that structured PBL on muscle physiology was associated with substantial short-term improvement in knowledge assessment scores and overwhelmingly positive student perception among first-year MBBS students who participated in the intervention. The magnitude of knowledge gain, coupled with the demonstrated research interest, suggests that early research exposure via group-based investigations was feasible to implement and was favorably received by participating students. However, the single-group observational design, self-selected sample, use of identical assessments, and absence of longer-term follow-up preclude causal inference regarding PBL's effectiveness relative to standard instruction.

Notwithstanding these preliminary findings, rigorously designed randomized comparative trials with concurrent control groups, extended follow-up periods, diverse institutional contexts, and mixed-methods outcome assessment are necessary to definitively establish PBL's relative efficacy, optimal implementation strategies, and conditions for sustainability within resource-constrained educational environments. Such investigations should prospectively evaluate the translation of expressed research interest into actual research participation, assess persistence of knowledge gains beyond the immediate post-intervention period, and examine implementation feasibility, cost-effectiveness, and scalability considerations. The present findings furnish sufficient preliminary evidence to warrant such comprehensive investigation.

## Appendices

Appendix A

Pre-Assessment Questionnaire for Project-Based Learning

Instructions: Please answer the following questions to the best of your knowledge. Provide your responses in a short-note format.

1. What is the length-tension relationship in skeletal muscle?

2. What is the strength-duration relationship?

3. What is muscle fatigue? Mention some factors affecting muscle fatigue.

4. What are the energy sources for muscle activity?

5. How can the level/intensity of exercise be graded on a physiological basis?

6. What is the respiratory quotient (RQ)?

7. What is resting metabolism of the body?

8. How does exercise affect the heart, lungs, and brain?

9. How do physical activity and exercise affect health?

10. Mention any diseases or conditions in which exercise capacity is limited.

Maximum score: 20 marks | Number of questions: 10 | Marks per question: 2 marks | Assessment format: Short-answer/short-note questions.

Appendix B

Student Feedback Questionnaire

Instructions: Please rate each statement/question using the following five-point Likert scale:

1 = None; 2 = Very little; 3 = Moderate; 4 = Much; 5 = A lot

1. How would you rate your overall experience of doing projects as part of the medical curriculum? (1 2 3 4 5)

2. How much knowledge of the subject matter (exercise physiology) have you gained as a result of participating in the projects? (1 2 3 4 5)

3. How much practical knowledge have you gained as a result of the project? (1 2 3 4 5)

4. Did you develop a specific interest in the subject matter as a result of the project? (1 2 3 4 5)

5. Did you become more interested in learning about research methods and techniques after completing the project? (1 2 3 4 5)

6. If opportunities for research projects in other topics or subjects were available, would you be interested in participating? (1 2 3 4 5)

Scoring: Each item is scored from 1 to 5, with higher scores indicating greater perceived benefit, interest, or satisfaction. The maximum possible total score is 30.
